# Biphalin, a Dimeric Enkephalin, Alleviates LPS-Induced Activation in Rat Primary Microglial Cultures in Opioid Receptor-Dependent and Receptor-Independent Manners

**DOI:** 10.1155/2017/3829472

**Published:** 2017-05-10

**Authors:** Katarzyna Popiolek-Barczyk, Anna Piotrowska, Wioletta Makuch, Joanna Mika

**Affiliations:** Department of Pain Pharmacology, Institute of Pharmacology, Polish Academy of Sciences, Kraków, Poland

## Abstract

Neuropathic pain is relatively less responsive to opioids than other types of pain, which is possibly due to a disrupted opioid system partially caused by the profound microglial cell activation that underlines neuroinflammation. We demonstrated that intrathecally injected biphalin, a dimeric enkephalin analog, diminished symptoms of neuropathy in a preclinical model of neuropathic pain in rats (CCI, chronic constriction injury of the sciatic nerve) at day 12 postinjury. Using primary microglial cell cultures, we revealed that biphalin did not influence cell viability but diminished NO production and expression of Iba1 in LPS-stimulated cells. Biphalin also diminished MOP receptor level, as well as pronociceptive mediators (iNOS, IL-1*β*, and IL-18) in an opioid receptor-dependent manner, and it was correlated with diminished p-NF-*κ*B, p-I*κ*B, p-p38MAPK, and TRIF levels. Biphalin reduced IL-6, IL-10, TNF*α*, p-STAT3, and p-ERK1/2 and upregulated SOCS3, TLR4, and MyD88; however, this effect was not reversed by naloxone pretreatment. Our study provides evidence that biphalin diminishes neuropathy symptoms, which might be partially related to reduced pronociceptive mediators released by activated microglia. Biphalin may be a putative drug for future pain therapy, especially for the treatment of neuropathic pain, when the lower analgesic effects of morphine are correlated with profound microglial cell activation.

## 1. Introduction

Although extensive studies have been performed to determine effective analgesics, opioids remain the gold standard in pain treatment. However, some animal and clinical studies have reported that neuropathic pain is not suppressed by low doses of opioids, which suppress nociceptive pain, and higher doses are necessary to obtain adequate analgesic effects [1–5]. Moreover, it was observed that chronic morphine treatment leads to the development of hypersensitivity and strong activation of glial cells [6–9]. Limited success in the therapy and the development of tolerance has led scientists to seek new drugs. In the present study, we used biphalin, a dimeric peptide [(Tyr-D-Ala-Gly-Phe-NH–)2] in which two enkephalin-type pharmacophores are connected “head-to-head” by a hydrazide bridge [10]. It was shown that biphalin has high affinity to mu opioid receptor (MOP) and delta opioid receptor (DOP) but lower affinity to kappa opioid receptor (KOP) [11, 12]. An antinociceptive character of biphalin was documented in an animal model of cancer pain [13], a semichronic colitis model [14], and in naïve animals [15, 16]. Biphalin exhibits 1000-fold greater analgesic potency than morphine [17, 18] and produces less side effects [17]. However, the analgesic effects of biphalin during neuropathic pain have not been explored.

A growing body of evidence indicates that neuropathic pain is associated with neuroinflammation, which is linked to the profound activation of numerous immunocompetent cells, such as microglia, within the central nervous system (CNS). The altered synaptic connectivity during neuropathy can be strongly modulated by microglia-derived immune factors. Pathological conditions within the CNS provoke rapid activation of microglia, which concern profound changes in morphology, proliferative potential, gene expression, and function. Additional elements of the activation process include the production of inflammatory mediators, such as cytokines (e.g., IL-1*β*, IL-6, IL-12, IL-15, IL-18, IFN*γ*, TNF*α*, CCL2, CCL3, CCL4, CCL5, and CCL7), enzymes (cyclooxygenase 2 (COX-2), inducible nitric oxide synthase (iNOS)), and cytotoxic compounds (e.g., nitric oxide (NO)) [19–28], which can act in deleterious or beneficial manners on surrounding cells, indirectly altering nociceptive signals. Given the crucial role of microglial activation and the associated neuroinflammation, it was suggested that this may be a new niche for the construction of an effective therapy against neuropathic pain.

Moreover, the results of our previous studies demonstrated that modulation of microglial cell activation not only diminished neuropathy but also strongly potentiated opioid (MOP and KOP) and nociceptin (NOP) receptor ligand analgesia [29, 30]. Several studies demonstrated that proinflammatory actions of morphine are related with microglial MOP receptor [31, 32]. The other interesting theory suggests that morphine is engaged in the microglial TLR4 (Toll-like receptor 4) signaling, which also seems to be responsible for the proinflammatory properties of this drug [32, 33]. However, there is no data showing a role of biphalin on TLR4, as well as MOP, receptor pathway activity.

Therefore, in the present study, we first examined the influence of biphalin on the syndrome of neuropathy in a preclinical model of neuropathic pain in rats (chronic constriction injury of the sciatic nerve, CCI). The next step of our work was to explore the possible beneficial action of biphalin on microglia in an in vitro model of LPS-induced microglial activation. We evaluated the effects of biphalin on microglial cell viability and nitric oxide production. Using western blot analysis, we explored the influence of biphalin on the marker of microglial cell activation (Iba1), TLR4, and MOP receptors levels as well as immune factors (iNOS, IL-1*β*, IL-18, COX-2, IL-6, IL-10, IFN*γ*, TNF*α*, and TIMP-1) and related intracellular signaling pathways (NF-*κ*B, I*κ*B, STAT3, SOCS3, p38MAPK, and ERK1/2) that underlie the development of neuroinflammation during neuropathy.

## 2. Materials and Methods

### 2.1. Animals

Male Wistar rats (300–350 g) were purchased from Charles River Laboratories (Hamburg, Germany). The animals were housed in cages lined with sawdust under a standard 12/12 h light/dark cycle (lights on at 08:00 h) with food and water available ad libitum. All experiments were performed according to the recommendations of IASP [34] and the NIH Guide for the Care and Use of Laboratory Animals and were approved by the II Local Bioethics Committee branch of the National Ethics Committee for Experiments on Animals based at the Institute of Pharmacology Polish Academy of Sciences (Cracow, Poland). Care was taken to minimize animal suffering and to reduce the number of animals used (3R policy).

### 2.2. Intrathecal (i.t.) Catheterization

The rats were chronically implanted with intrathecal (i.t.) catheters according to Yaksh and Rudy [35] under pentobarbital anesthesia (60 mg/kg; intraperitoneal (i.p.)). The intrathecal catheter consisted of polyethylene tubing that was 12 cm long (PE10, Intramedic; Clay Adams, Parsippany, NJ) with an outer diameter of 0.4 mm and a dead space of 10 *μ*L. The catheter was sterilized by immersion in 70% (v/v) ethanol and fully flushed with sterile water before insertion. The rats were placed on a stereotaxic table (David Kopf Instruments, Tujunga, CA), and an incision was made in the atlanto-occipital membrane. The catheter (7.8 cm of its length) was carefully introduced into the subarachnoid space at the rostral level of the spinal cord lumbar enlargement (L4-L5). After implantation, the first injection of 10 *μ*L water was performed slowly, and the catheter was tightened. The rats were monitored for physical impairments, and those showing motor deficits were excluded from further study. Animals were allowed 1 week of recovery after the surgery before the experiment began. Substances were injected slowly (1-2 min) in a volume of 5 *μ*L through the i.t. catheter and were followed by 10 *μ*L water.

### 2.3. Sciatic Nerve Surgery

Chronic constriction injury (CCI) was produced in the rats according to Bennett and Xie [36]. The procedure was performed on the 7th day after catheter implantation under pentobarbital anesthesia (60 mg/kg; i.p.). The biceps femoris and the gluteus superficialis were separated, and the right sciatic nerve was exposed. The injury was induced by tying four loose ligatures (4/0 silk) with 1 mm spacing around the sciatic nerve. The brief twitch of muscles in the respective hind limb during ligation prevented the application of the ligatures from being strong. As a control group, we used naïve animals (no procedure was conducted on those rats) and the sham-operated group (under anesthesia surgery up to sciatic nerve exposure without ligation). Only CCI-operated rats developed long-lasting tactile and thermal hypersensitivity.

### 2.4. Behavioral Tests

#### 2.4.1. Tactile Hypersensitivity (von Frey Test)

In animal models [37] and in patients [38], touch-evoked pain is a hallmark of neuropathy; therefore, development of tactile hypersensitivity was assessed in our studies. Tactile hypersensitivity was measured in the rats subjected to CCI 12 days after surgery by the use of an automatic von Frey apparatus (Dynamic Plantar Aesthesiometer, Ugo Basile, Italy). The rats were placed in plastic cages with a wire net floor 5 min before the experiment. The von Frey filament (the strength of stimuli ranged from 0.5 g to 26 g) was applied to the midplantar surface of the hind foot, and measurements were taken automatically. All experiments were conducted 30 min after the i.t. administration of biphalin or vehicle.

#### 2.4.2. Thermal Hypersensitivity (Cold Plate Test)

Thermal hypersensitivity was assessed using the cold plate test (Cold/Hot Plate Analgesia Meter, Columbus Instruments, USA) as has been described previously [37]. The temperature of the cold plate was maintained at 5°C, and the cut-off latency was 30 s. The rats were placed on the cold plate, and the time until the lifting of the hind foot was recorded. The injured foot was the first to react in every case.

### 2.5. Drug Administration

Biphalin hydrochloride (PubChem CID: 5487663) was a kind gift from Professor Andrzej Lipkowski (Mossakowski Medical Research Centre PAS, Poland). The behavioral tests were performed 30 min (von Frey) and 35 min (cold plate) after biphalin administration on day 12 post-CCI.

### 2.6. Microglial Cell Cultures and Treatments

Primary cultures of microglial cells were prepared from the cerebral cortices of 1-day-old Wistar rat pups, as previously described [25]. Briefly, isolated cells were plated on poly-L-lysine-coated 75 cm^2^ culture flasks at a density of 3 × 10^5^ cells/cm^2^ in a culture medium that consisted of Dulbecco's modified Eagle's medium (DMEM)/GlutaMAX/high glucose (4.5 g/L) (Gibco, USA) supplemented with heat-inactivated 10% fetal bovine serum (FBS) (Gibco, USA), 100 U/mL penicillin, and 0.1 mg/mL streptomycin (Gibco, USA), and the cultures were maintained at 37°C and 5% CO_2_. The culture medium was changed after 4 days. On day 9, the loosely adherent microglial cells were recovered by mild shaking in a horizontal shaker (80 rpm for 1 h and 100 rpm for 15 min) and cell viability was determined via the trypan blue exclusion method using a TC20-automated cell counter (Bio-Rad, Poland). Microglial cells were suspended in a culture medium and plated at final densities of 1.2 × 10^6^, 2 × 10^5^, and 4 × 10^4^ cells onto 6-well, 24-well, and 96-well plates, respectively. Adherent cells were incubated for 48 h in culture medium before being used for the analyses. Cell specificity was determined by western blot analysis using Iba-1 (a microglial marker, Santa Cruz) and GFAP (an astrocyte marker, Santa Cruz). We obtained highly homogeneous microglial populations (cultured cells were more than 97% positive for Iba-1) [39].

Opioid peptide biphalin hydrochloride was dissolved in aqua proinjection. Primary microglial cell cultures were treated with biphalin (0.1, 1, 10, or 20 *μ*M) for 30 min and then 1 h or 24 h with vehicle (PBS) or LPS (100 ng/mL) (lipopolysaccharide from *Escherichia coli* 0111:B4; Sigma-Aldrich, USA). In antagonist experiments, the opioid receptor antagonist naloxone (0.1 *μ*M; Tocris, UK) was added 30 min before biphalin treatment.

### 2.7. Cell Viability Assay

The cell viability after biphalin treatment alone and after LPS administration was determined by a tetrazolium salt 3-[4,5-dimethylthiazol-2-yl]-2,5-diphenyltetrazolium bromide assay (MTT, Sigma-Aldrich, Germany). After 24 h of treatment with different concentrations of biphalin (0.1, 1, 10, and 20 *μ*M) with or without LPS (100 ng/mL), MTT (at a concentration of 0.15 mg/mL) was added to each well, and the cells were incubated for 2 h at 37°C. Next, the culture medium was discarded, and 0.1 M HCl in isopropanol was added to dissolve the formazan dye. The absorbance values were measured using a Multiskan Spectrum apparatus at 570 nm. The data were normalized to the absorbance in the control group (vehicle-treated cells) and expressed as a percentage of the control ± SEM.

### 2.8. Griess Assay

To quantify aqueous nitrite concentrations, the Griess method was used. The method involves a colorimetric measurement of the concentration of nitrite ions (NO^2−^), a stable, nonvolatile breakdown product of nitric oxide (NO). The medium collected from above the tested cells (50 *μ*L) was transferred to 96-well plates in triplicate. Griess A reagent (1% sulfanilamide in 5% phosphoric acid) was added to the tested medium, and after incubation (10 min, RT), Griess B reagent (0.1%, dihydrochloride N-(1-naphthyl)-ethylenediamine) was added. The absorbance of the samples was read at *λ* = 540 nm using a Multiskan Spectrum apparatus. The data were normalized to the absorbance of the control group (vehicle-treated cells) and are expressed as a percentage of released nitric oxide ± SEM.

### 2.9. Western Blot

Cell lysates were collected in RIPA buffer with a protease inhibitor cocktail (Sigma-Aldrich, USA). The mixtures were cleared by centrifugation (14000 ×g for 30 min at 4°C). Then, samples containing 10 *μ*g protein were heated in a loading buffer (4x Laemmli Buffer, Bio-Rad, Poland) for 5 min at 98°C and were loaded on 4–15% or 7.5% Criterion™ TGX™ precast polyacrylamide gels (Bio-Rad, Poland). The proteins were transferred to Immune-Blot PVDF membranes (Bio-Rad, Poland) with semidry transfer (30 min, 25 V). The membranes were blocked for 1 h at RT using 5% nonfat, dry milk (Bio-Rad, Poland) in Tris-buffered saline with 0.1% Tween-20 (TBST). The membranes were then washed in TBST (4 ×5 min) and incubated overnight at 4°C with primary antibodies. The following primary antibodies were used during the studies: rabbit polyclonal TLR4 (Santa Cruz) 1 : 500; MyD88 (Novus) 1 : 500; TRIF (Novus) 1 : 500; MOP (Abcam) 1 : 500; Iba1 (Santa Cruz) 1 : 500; IL-1*β* (Abcam) 1 : 1000; IL-18 (R&D Systems) 1 : 1000; IL-6 (Invitrogen) 1 : 500; iNOS (Santa Cruz) 1 : 500; IL-10 (Invitrogen) 1 : 500; IFN*γ* (Cell Signaling) 1 : 500; TNF*α* (Cell Signaling) 1 : 500; TIMP-1 (Novus Biologicals) 1 : 1000; COX-2 (Abcam) 1 : 1000; NLRP3 (Santa Cruz) 1 : 250; SOCS3 (Cell Signaling) 1 : 500; p-p38 MAPK (Cell Signaling) 1 : 1000; p38 MAPK (Cell Signaling) 1 : 1000; p-ERK1/2 (Cell Signaling) 1 : 1000; ERK1/2 (Cell Signaling) 1 : 1000; p-NF-*κ*B (Santa Cruz) 1 : 500; NF-*κ*B (Santa Cruz) 1 : 500; p-I*κ*B (Santa Cruz) 1 : 500; I*κ*B (Santa Cruz) 1 : 500; p-STAT3 (Cell Signaling) 1 : 500; STAT3 (Cell Signaling) 1 : 500, and mouse polyclonal GAPDH (Millipore) 1 : 5000. Then, the membranes were incubated for 1 h in horseradish peroxidase-conjugated anti-rabbit or anti-mouse secondary antibodies (1 : 5000). All primary and secondary antibodies were diluted using a SignalBoost™ Immunoreaction Enhancer Kit (Merck Millipore Darmstadt, Germany). The membranes were washed (4 × 5 min) with TBST. The Clarity™ Western ECL Substrate (Bio-Rad, Poland) was used to detect immunocomplexes, which were then visualized using a Fujifilm LAS-4000 FluorImager system. Blots were washed 2 times for 5 min each in TBST, stripped using Restore Western Blot Stripping Buffer (Thermo Scientific), washed (2 × 5 min) in TBST, blocked, and reprobed with an antibody against GAPDH as an internal loading control. The relative levels of immunoreactivity were quantified densitometrically using Fujifilm Multi Gauge software.

### 2.10. Immunocytochemical Analysis

We used commercially available specific anti-MOP and anti-OX/42 antibodies. Microglial cells were fixed for 20 minutes in 4% paraformaldehyde in a 0.1 M phosphate buffer (pH 7.4) and then incubated with primary antibodies (rabbit anti-MOP 1 ∶ 400, mouse anti-OX/42 1 ∶ 500; Serotec) for 2 days at 4°C. After three washes in PB, double immunofluorescence was revealed by incubation for 2 h in the appropriate fluorochrome-conjugated secondary antibody (Alexa Fluor546 donkey anti-rabbit 1 : 500, Alexa Fluor488 donkey anti-mouse 1 : 500), diluted in 5% NDS. Sections were washed with PB and then coverslipped with an Aquatex mounting medium (Merck, Darmstadt, Germany). Sections without primary antibodies were used as negative controls.

### 2.11. Statistical Analyses

**Figure 1:**
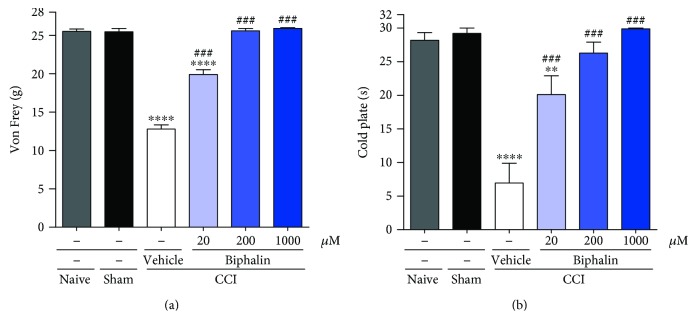
The effect of a single intrathecal administration of biphalin at a dose ranging from 20 to 1000 *μ*M on mechanical (a) and thermal (b) hypersensitivity as measured by von Frey and cold plate tests on day 12 after CCI. The results are presented as the means ± SEM (5–9 rats per group). The results were statistically evaluated using one-way analysis of variance (ANOVA) followed by Bonferroni's post hoc test to assess differences between treatment groups. ^∗∗∗∗^*P* < 0.0001 and ^∗∗^*P* < 0.01 indicate differences compared to sham-operated rats; ^###^*P* < 0.001 indicates differences compared to the vehicle-treated CCI-exposed rats. The cut-off latency was 26 g for von Frey test and 30 s for cold plate test.

The behavioral data (Figure 1) are presented as the mean ± SEM of 6–9 rats per group. The results of the experiments were statistically evaluated using one-way analysis of variance (ANOVA) followed by Bonferroni's test for multiple comparisons. Significant differences between sham-operated rats and CCI-exposed rats are indicated by ^∗∗^*P* < 0.01 and ^∗∗∗∗^*P* < 0.0001. Significant differences between vehicle-treated CCI-exposed rats and biphalin-treated CCI-exposed rats are indicated by ^###^*P* < 0.001.

**Figure 2:**
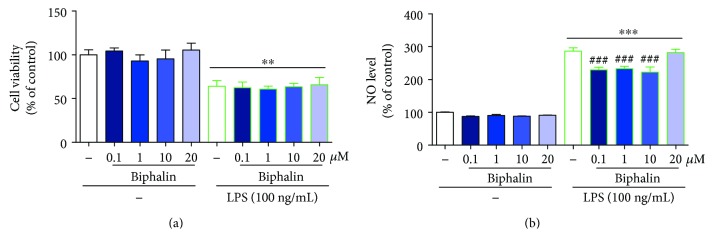
The influence of biphalin on cell viability (a) and nitric oxide secretion (b) in vehicle- and LPS-treated primary microglial cells. Biphalin (0.1, 1, 10, and 20 *μ*M) was added to the culture medium 30 min before LPS (100 ng/mL) treatment, and then cells were cultured for 24 h. The results are presented as a percentage of control (vehicle-treated cells) as the mean ± SEM of 3-4 independent experiments. The results were statistically evaluated using one-way analysis of variance (ANOVA) followed by Bonferroni's post hoc test to assess differences between the treatment groups. Significant differences in comparison with those of the control group (vehicle-treated cells) are indicated by ^∗∗^*P* < 0.01 and ^∗∗∗^*P* < 0.001; differences between LPS-treated and biphalin-treated cells are indicated by ^###^*P* < 0.001.

The results of the cell viability and Griess assays (Figure 2) are presented as a percentage of control (vehicle-treated cells) as the mean ± SEM of 3-4 independent experiments. The results were statistically evaluated using one-way analysis of variance (ANOVA) with Bonferroni's post hoc test to assess the differences between the treatment groups. Significant differences in comparison with those of the control group (vehicle-treated cells) are indicated by ^∗∗^*P* < 0.01 and ^∗∗∗^*P* < 0.001; differences between LPS-treated and biphalin-treated cells are indicated by ^###^*P* < 0.001.

**Figure 3:**
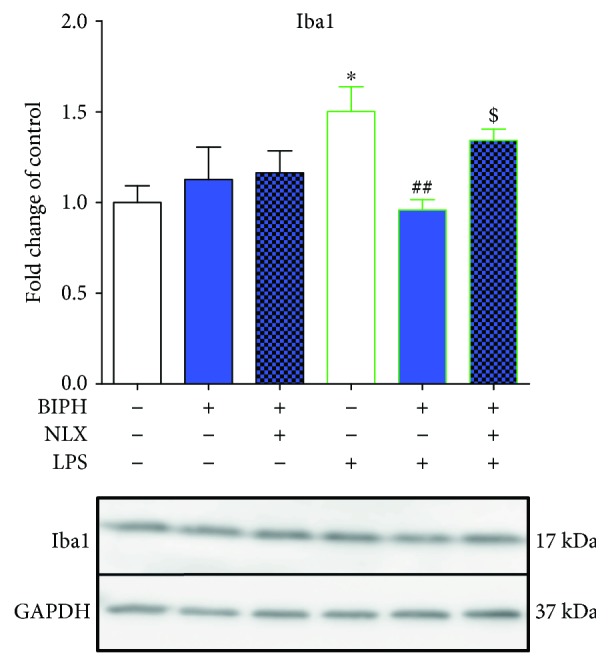
The influence of biphalin on Iba1 protein levels in vehicle- and LPS-treated primary microglial cells. Microglial cells were treated with biphalin (BIPH; 10 *μ*M) for 30 min and then with LPS (100 ng/mL) for 24 h. Naloxone (NLX; 0.1 *μ*M) was added 30 min before biphalin. The data are presented as the fold change compared with the control group (vehicle-treated cells) as the mean ± SEM of 3-4 independent experiments. The results were statistically evaluated using one-way analysis of variance (ANOVA) followed by Bonferroni's post hoc test to assess the differences between the treatment groups. Significant differences in comparison with those of the control group (vehicle-treated cells) are indicated by ^∗^*P* < 0.05; differences between LPS-treated and biphalin- or biphalin- and naloxone-treated cells are indicated by ^##^*P* < 0.01; differences between biphalin-treated and biphalin- and naloxone-treated cells are indicated by ^$^*P* < 0.05.

**Figure 4:**
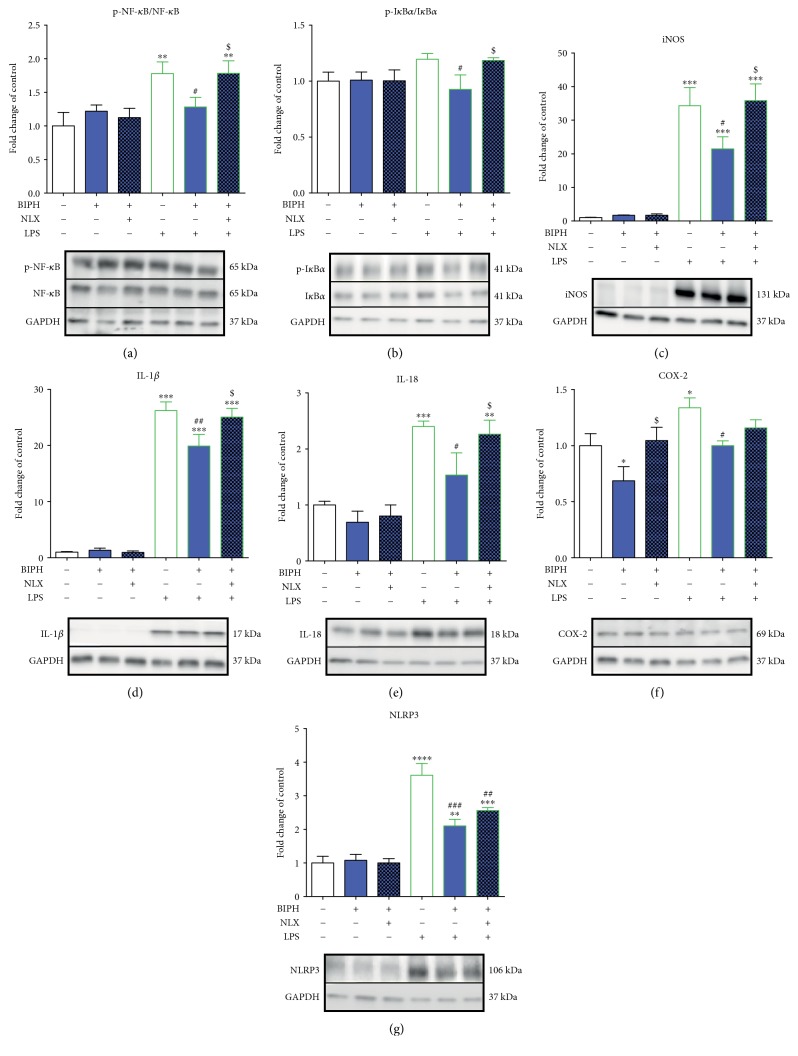
The influence of biphalin on NF-*κ*B (a) and I*κ*B (b) phosphorylation and iNOS (c), IL-1*β* (d), IL-18 (e), COX-2 (f), and NLRP3 (g) protein levels in vehicle- and LPS-treated primary microglial cells. Microglial cells were treated with biphalin (BIPH; 10 *μ*M) for 30 min and then with LPS (100 ng/mL) for 1 h (a, b) or 24 h (c, d, e, f, g). Naloxone (NLX; 0.1 *μ*M) was added 30 min before biphalin. The data are presented as the fold change compared with the control group (vehicle-treated cells) as the mean ± SEM of 3–6 independent experiments. The results were statistically evaluated using one-way analysis of variance (ANOVA) followed by Bonferroni's post hoc test to assess the differences between the treatment groups. Significant differences in comparison with those of the control group (vehicle-treated cells) are indicated by ^∗^*P* < 0.05, ^∗∗^*P* < 0.01, ^∗∗∗^*P* < 0.001, and ^∗∗∗∗^*P* < 0.0001; differences between LPS-treated and biphalin- or biphalin- and naloxone-treated cells are indicated by ^#^*P* < 0.05, ^##^*P* < 0.01, and ^###^*P* < 0.001; differences between biphalin-treated and biphalin- and naloxone-treated cells are indicated by ^$^*P* < 0.05.

**Figure 5:**
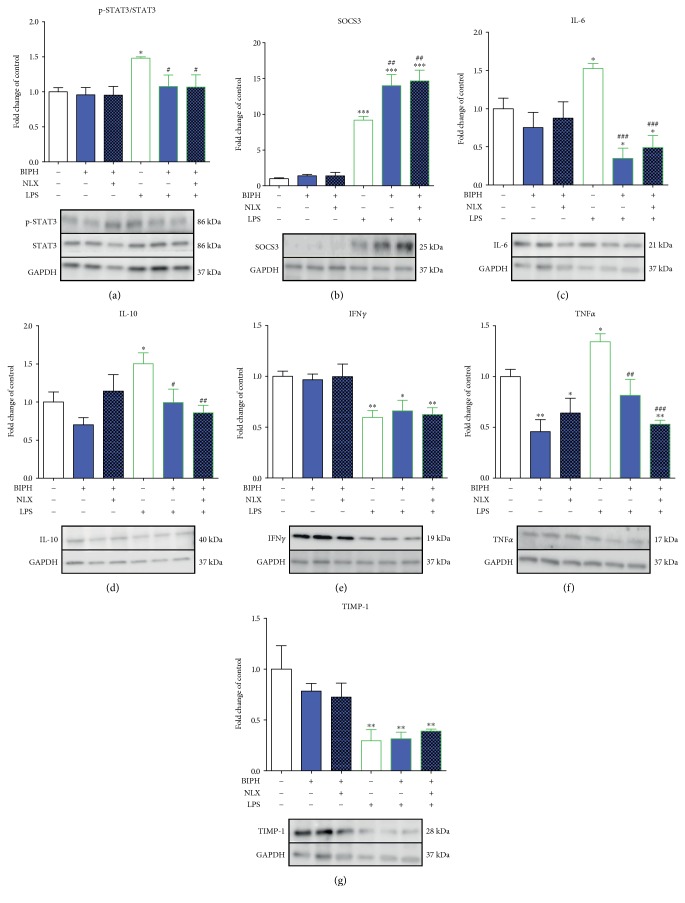
The influence of biphalin on STAT3 (a) phosphorylation and SOCS3 (b), IL-6 (c), IL-10 (d), IFN*γ* (e), TNF*α* (f), and TIMP-1 (g) protein levels in vehicle- and LPS-treated primary microglial cells. Microglial cells were treated with biphalin (BIPH; 10 *μ*M) for 30 min and then with LPS (100 ng/mL) for 1 h (a) or 24 h (b, c, d, e, f, g). Naloxone (NLX; 0.1 *μ*M) was added 30 min before biphalin. The data are presented as the fold change compared with the control group (vehicle-treated cells) as the mean ± SEM of 3-4 independent experiments. The results were statistically evaluated using one-way analysis of variance (ANOVA) followed by Bonferroni's post hoc test to assess differences between the treatment groups. Significant differences in comparison with those of the control group (vehicle-treated cells) are indicated by ^∗^*P* < 0.05, ^∗∗^*P* < 0.01, and ^∗∗∗^*P* < 0.001; differences between LPS-treated and biphalin- or biphalin- and naloxone-treated cells are indicated by ^#^*P* < 0.05, ^##^*P* < 0.01, and ^###^*P* < 0.001.

The results of the western blot (Figures 3, 4, 5, 7, and 8) are presented as the fold change compared with the control group (vehicle-treated cells) as the mean ± SEM of 3–6 independent experiments. The results were statistically evaluated using one-way analysis of variance (ANOVA) with Bonferroni's post hoc test to assess the differences between the treatment groups. Significant differences in comparison with those of the control group (vehicle-treated cells) are indicated by ^∗^*P* < 0.05, ^∗∗^*P* < 0.01, ^∗∗∗^*P* < 0.001, and ^∗∗∗∗^*P* < 0.0001; differences between LPS-treated and biphalin- or biphalin- and naloxone-treated cells are indicated by ^#^*P* < 0.05, ^##^*P* < 0.01, and ^###^*P* < 0.001; differences between biphalin-treated and biphalin- and naloxone-treated cells are indicated by ^$^*P* < 0.05 and ^$$^*P* < 0.01.

All graphs and analyses were prepared using GraphPad Prism5.

## 3. Results

### 3.1. The Influence of Biphalin on Neuropathic Pain Symptoms in Rats

In the first set of experiments, we tested the analgesic effects of biphalin in a preclinical model of neuropathic pain in rats 12 days after injury. The administration of biphalin (20, 200, and 1000 *μ*M; i.t.) attenuated the development of tactile hypersensitivity as measured by von Frey test 30 min after drug injection as compared to the vehicle-treated CCI-exposed rats (12.78 g ± 0.55 versus 19.88 g ± 0.63, 25.58 g ± 0.32, and 25.91 g ± 0.09) (Figure 1(a)).

In CCI-exposed rats, the administration of biphalin (20, 200, and 1000 *μ*M; i.t.) also attenuated the development of thermal hypersensitivity as measured by cold plate test 35 min after drug administration as compared to the vehicle-treated CCI-exposed animals (6.93 s ± 2.97 versus 20.11 s ± 2.81, 26.27 s ± 1.67, and 29.90 s ± 0.11) (Figure 1(b)).

We did not observed any significant changes in hypersensitivity between sham-operated and naïve animals (Figures 1(a) and 1(b)).

### 3.2. The Influence of Biphalin on Cell Viability and Nitric Oxide Secretion in Vehicle- and LPS-Treated Microglial Cells

Using primary microglial cell cultures, we examined the effect of different doses of biphalin (0.1, 1, 10, and 20 *μ*M) on the cell viability. As shown in Figure 2, 24 h treatment did not change microglial cell viability, as measured by the MTT reduction assay (Figure 2(a)). LPS treatment (100 ng/mL) resulted in a lower cell viability in primary microglial cultures compared to vehicle-stimulated control (100 ± 5.7% versus 64.03 ± 6.5%) (Figure 2(a)). Biphalin was added to the culture medium 30 min before LPS treatment. None of the selected doses of biphalin modulated LPS-induced cell death (Figure 2(a)).

In the next set of experiments, we analyzed the influence of different doses of biphalin (0.1, 1, 10, and 20 *μ*M) on the secretion of nitric oxide (NO) by microglial cells. None of the tested doses of biphalin influenced NO production in vehicle-treated cells (Figure 2(b)). LPS administration (100 ng/mL) significantly potentiated NO release by microglia compared to vehicle-stimulated control (100 ± 1.13% versus 286.38 ± 10.37%). A decrease in the secretion of NO was observed after biphalin treatment at doses of 0.1, 1, and 10 *μ*M compared to LPS-stimulated control (222.34 ± 15.86%, 233.19 ± 7.31%, and 229.36 ± 8.30%, resp., versus 286.38 ± 10.37%). Based on the results obtained from the above studies, we decided to perform subsequent experiments using biphalin at the dose of 10 *μ*M.

We did not observed any changes in expression of microglial cell activation marker (Iba1) after biphalin [10 *μ*M] treatment in control cells (Figure 3). However, in LPS-stimulated cells, biphalin reduced upregulated level of Iba1 (1.50 ± 0.14 versus 0.96 ± 0.06) (Figure 3).

### 3.3. The Influence of Biphalin on NF-*κ*B and I*κ*B Phosphorylation and iNOS, IL-1*β*, IL-18, COX-2, and NLRP3 Expression in Vehicle- and LPS-Treated Microglial Cells

LPS stimulation significantly increased the level of phosphorylation of nuclear factor-*κ*B (NF-*κ*B) compared to the vehicle-treated group (1 ± 0.2 versus 1.78 ± 0.17) (Figure 4(a)). Significant downregulation of p-NF-*κ*B was observed after biphalin treatment in LPS-stimulated cells (1.28 ± 0.14), and this effect was reversed after naloxone pretreatment (1.78 ± 0.18). There were no changes in NF-*κ*B activation levels after treatment with compounds in vehicle-treated cells (Figure 4(a)).

In LPS-stimulated cells, biphalin significantly diminished level of p-I*κ*B (nuclear factor-*κ*B inhibitor *α*) (0.92 ± 0.13 versus 1.19 ± 0.05) (Figure 4(b)). Biphalin action was reversed by naloxone pretreatment (1.18 ± 0.02) (Figure 4(b)). No changes in p-I*κ*B protein level was observed in vehicle-treated cells stimulated with biphalin alone or with naloxone (Figure 4(b)).

The expression of iNOS (1.0 ± 0.08 versus 34.27 ± 5.39) was significantly elevated after LPS treatment compared to control cells (Figure 4(c)), and this effect was attenuated by biphalin pretreatment (21.45 ± 3.63). Naloxone significantly diminished biphalin effects and restored the iNOS level nearly to that of the LPS-treated group (35.84 ± 4.97). No changes were observed in vehicle-treated cells after stimulation with compounds (Figure 4(c)).

The level of proinflammatory interleukin 1*β* (IL-1*β*) (1 ± 0.13 versus 26.24 ± 1.55) was significantly potentiated after LPS treatment compared to control cells (Figure 4(d)). Biphalin pretreatment attenuated LPS-induced activation of IL-1*β* (19.91 ± 2.07). Naloxone significantly diminished biphalin effects (25.05 ± 1.56). No changes in IL-1*β* expression were observed after treatment with compounds in vehicle-treated cells (Figure 4(d)).

The IL-18 protein level was upregulated in microglia from 1.0 ± 0.07 to 2.4 ± 0.09 (Figure 4(e)) in LPS-stimulated cells compared with vehicle-treated control cells. The elevated level of IL-18 was diminished by biphalin (1.53 ± 0.39) in LPS-stimulated cells, and this effect was reversed by naloxone pretreatment (2.26 ± 0.25). There were no changes in IL-18 expression in vehicle-treated cells after biphalin alone or with naloxone (Figure 4(e)).

The protein level of COX-2 was elevated after LPS treatment compared to vehicle-treated control (1.0 ± 0.11 versus 1.34 ± 0.09) (Figure 4(f)). The high level of COX-2 was diminished to the control level (1.0 ± 0.04) after biphalin treatment. Naloxone pretreatment slightly diminished biphalin action (1.16 ± 0.07), although this effect was not significant. Moreover, biphalin diminished COX-2 protein level in vehicle-treated cells (0.69 ± 0.13), and this action was reversed by naloxone pretreatment (1.04 ± 0.12) (Figure 4(f)).

The level of inflammasome NLRP3 protein level was upregulated after LPS stimulation compared with the vehicle-treated control (1.0 ± 0.19 versus 3.61 ± 0.35) (Figure 4(g)). This elevated level was diminished by biphalin (2.11 ± 0.19) in LPS-stimulated cells (Figure 4(g)). Naloxone did not significantly modulate biphalin action, although we observed a strong growing trend after pretreatment with this antagonist (2.56 ± 0.08) (Figure 4(g)). There were no changes in NLRP3 expression in vehicle-treated cells after stimulation with biphalin alone or with naloxone (Figure 4(g)).

### 3.4. The Influence of Biphalin on STAT3 Phosphorylation and SOCS3, IL-6, IL-10, TNF*α*, IFN*γ*, and TIMP-1 Expression in Vehicle- and LPS-Treated Microglial Cells

LPS stimulation significantly increased the level of phosphorylation of STAT3 compared to the vehicle-treated group (1 ± 0.06 versus 1.48 ± 0.02) (Figure 5(a)). Biphalin significantly downregulated p-STAT3 levels in LPS-stimulated cells (1.07 ± 0.16), although this effect was not modulated by naloxone pretreatment (1.06 ± 0.18). There were no changes in STAT3 phosphorylation after treatment with compounds in vehicle-treated cells (Figure 5(a)).

The level of SOCS3 was significantly upregulated after LPS treatment compared to controls (1 ± 0.12 versus 9.19 ± 0.53) (Figure 5(b)). Biphalin pretreatment potentiated LPS-induced expression of SOCS3 protein (14.01 ± 1.54). Moreover, naloxone pretreatment did not change the effect of biphalin (14.66 ± 1.5). No changes in the expression of SOCS3 were observed after treatment with compounds in vehicle-treated cells (Figure 5(b)).

LPS treatment induced significant expression of IL-6 compared to vehicle-treated cells (1.0 ± 0.14 versus 1.53 ± 0.06) (Figure 5(c)). In LPS-stimulated cells, biphalin diminished the level of IL-6 (0.35 ± 0.13) (Figure 5(c)). This biphalin action was maintained even for naloxone pretreatment (0.49 ± 0.16) (Figure 5(c)). No changes in IL-6 protein level were observed in vehicle-treated cells stimulated with the tested compounds (Figure 5(b)).

The level of antinociceptive IL-10 was elevated after LPS stimulation compared to controls (1.0 ± 0.13 versus 1.5 ± 0.14) (Figure 5(d)). Biphalin pretreatment attenuated LPS-induced activation of IL-10 (0.99 ± 0.18), and this action was not reversed by naloxone (0.86 ± 0.09). No changes in IL-10 expression were observed after treatment with compounds in vehicle-treated cells, although we observed a decreasing trend after biphalin stimulation (Figure 5(d)).

The expression of IFN*γ* (1.0 ± 0.05 versus 0.59 ± 0.06) was significantly downregulated after LPS treatment compared to controls, and this effect was not changed by biphalin alone or biphalin with naloxone (Figure 5(e)). In addition, no changes were observed in vehicle-treated cells after compound stimulation (Figure 5(e)).

The TNF*α* protein level was upregulated in the microglia (1.0 ± 0.07 to 1.34 ± 0.08) (Figure 5(f)) in LPS-stimulated cells compared with vehicle-treated control cells. The elevated level of TNF*α* was diminished by biphalin (0.81 ± 0.16) in LPS-stimulated cells, and this effect was not reversed by naloxone pretreatment (0.53 ± 0.03) (Figure 5(f)). Moreover, biphalin significantly diminished TNF*α* protein levels in vehicle-treated cells (0.46 ± 0.11), and naloxone stimulation also did not change biphalin effects (0.64 ± 0.14) (Figure 5(f)).

The protein level of TIMP-1 was diminished after LPS treatment compared to vehicle-treated control cells (1.0 ± 0.23 versus 0.29 ± 0.11) (Figure 5(g)). Biphalin alone treatment (0.32 ± 0.07), as well as biphalin with naloxone (0.39 ± 0.02), did not change the LPS effect. No significant changes were observed in vehicle-treated cells after stimulation with compounds (Figure 5(g)).

### 3.5. The Influence of Biphalin on p38 and ERK1/2 Phosphorylation in Vehicle- and LPS-Treated Microglial Cells

**Figure 6:**
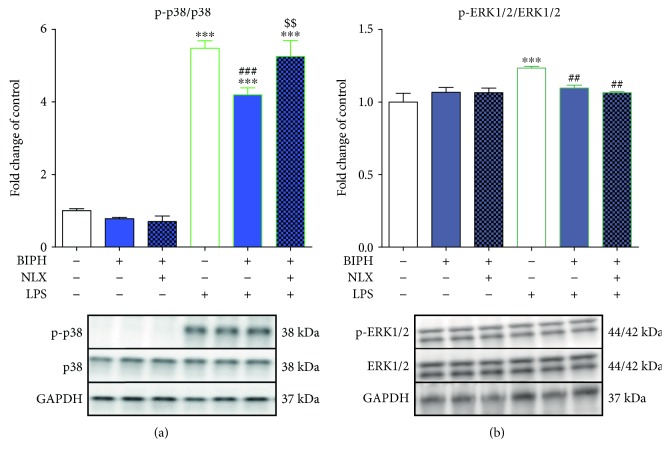
The influence of biphalin on p38 (a) and ERK1/2 (b) phosphorylation in vehicle- and LPS-treated primary microglial cells. Microglial cells were treated with biphalin (BIPH; 10 *μ*M) for 30 min and then with LPS (100 ng/mL) for 1 h (a, b). Naloxone (NLX; 0.1 *μ*M) was added 30 min before biphalin. The data are presented as the fold change compared with the control group (vehicle-treated cells) as the mean ± SEM of 3-4 independent experiments. The results were statistically evaluated using one-way analysis of variance (ANOVA) followed by Bonferroni's post hoc test to assess differences between the treatment groups. Significant differences in comparison with those of the control group (vehicle-treated cells) are indicated by ^∗∗∗^*P* < 0.001; differences between LPS-treated and biphalin- or biphalin- and naloxone-treated cells are indicated by ^##^*P* < 0.01 and ^###^*P* < 0.001; differences between biphalin-treated and biphalin- and naloxone-treated cells are indicated by ^$$^*P* < 0.01.

The level of phosphorylation of p38 (p-p38) compared to the vehicle-treated group after LPS stimulation was significantly increased (1 ± 0.06 versus 5.48 ± 0.2) (Figure 6(a)). In cells pretreated with biphalin, the level of p-p38 was diminished (4.19 ± 0.19). Moreover, stimulation with naloxone restored biphalin effects (5.25 ± 0.44). None of the used compounds modulated the p-p38 level in vehicle-treated cells (Figure 6(a)).

Stimulation of microglial cells with LPS significantly elevated the level of ERK1/2 phosphorylation (p-ERK1/2) compared to control cells (1 ± 0.06 versus 1.23 ± 0.01) (Figure 6(b)). Biphalin pretreatment reduced LPS-induced ERK phosphorylation (1.09 ± 0.02), although this effect was not modulated by naloxone (1.06 ± 0.01). No changes in p-ERK1/2 level was observed after treatment with compounds in vehicle-treated cells (Figure 6(b)).

### 3.6. The Influence of Biphalin on TLR4, MyD88, and TRIF Expression in Vehicle- and LPS-Treated Microglial Cells

**Figure 7:**
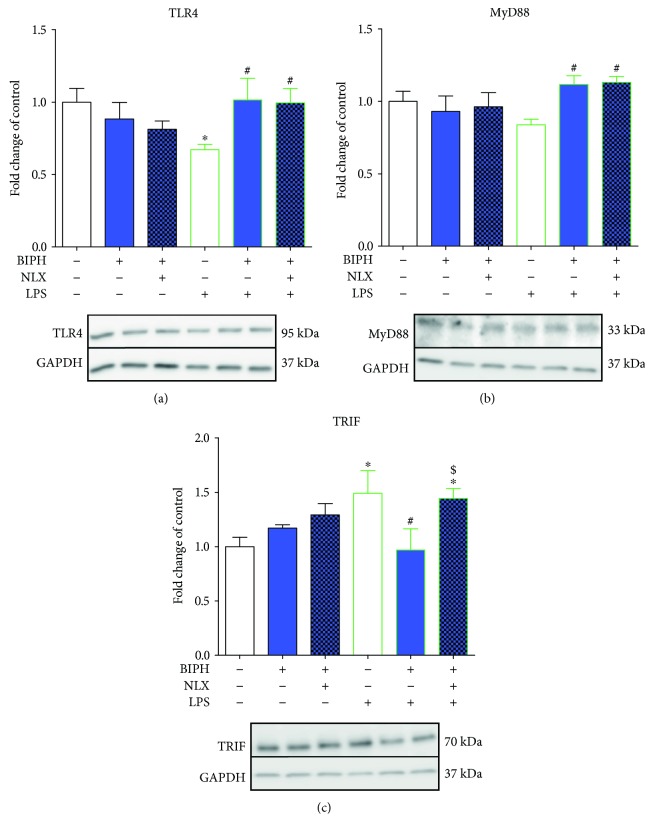
The influence of biphalin on TLR4 (a), MyD88 (b), and TRIF (c) protein levels in vehicle- and LPS-treated primary microglial cells. Microglial cells were treated with biphalin (BIPH; 10 *μ*M) for 30 min and then with LPS (100 ng/mL) 24 h (a, b, c). Naloxone (NLX; 0.1 *μ*M) was added 30 min before biphalin. The data are presented as the fold change compared with the control group (vehicle-treated cells) as the mean ± SEM of 3-4 independent experiments. The results were statistically evaluated using one-way analysis of variance (ANOVA) followed by Bonferroni's post hoc test to assess the differences between the treatment groups. Significant differences in comparison with those of the control group (vehicle-treated cells) are indicated by ^∗^*P* < 0.05; differences between LPS-treated and biphalin- or biphalin- and naloxone-treated cells are indicated by ^#^*P* < 0.05; differences between biphalin-treated and biphalin- and naloxone-treated cells are indicated by ^$^*P* < 0.05.

The TLR4 protein level was downregulated in microglia from 1.0 ± 0.09 to 0.67 ± 0.04 (Figure 7(a)) in LPS-stimulated cells compared with control cells. In cells pretreated with biphalin, protein of TLR4 was restored to control level (1.02 ± 0.15). The effect of biphalin was not reversed by naloxone pretreatment (0.99 ± 0.1) (Figure 7(a)).

LPS stimulation diminished protein level of MyD88 (1.0 ± 0.07 versus 0.84 ± 0.04 compared to control cells); however, those changes were not statistically significant (Figure 7(b)). In LPS-stimulated cells, biphalin restored MyD88 level to control (1.11 ± 0.06), although this effect was not modulated by naloxone (1.13 ± 0.04).

Stimulation of microglial cells with LPS significantly elevated the level of TRIF compared to control cells (1 ± 0.09 versus 1.49 ± 0.2) (Figure 7(c)). Biphalin reduced TRIF expression to control level (0.97 ± 0.19). The effect of biphalin was restored to LPS-treated control after naloxone pretreatment (1.08 ± 0.09). No changes in protein level of TRIF was observed after treatment with compounds in vehicle-treated cells (Figure 7(c)).

### 3.7. The Influence of Biphalin on MOP Expression in Vehicle- and LPS-Treated Microglial Cells

**Figure 8:**
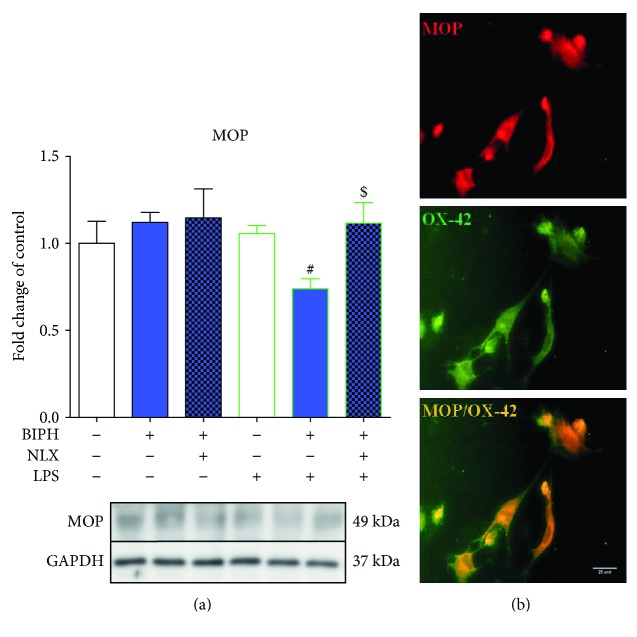
The influence of biphalin on MOP (a) protein levels in vehicle- and LPS-treated primary microglial cells and the immunocytochemical localization of MOP (b). (a) Microglial cells were treated with biphalin (BIPH; 10 *μ*M) for 30 min and then with LPS (100 ng/mL) 24 h. Naloxone (NLX; 0.1 *μ*M) was added 30 min before biphalin. The data are presented as the fold change compared with the control group (vehicle-treated cells) as the mean ± SEM of 4 independent experiments. The results were statistically evaluated using one-way analysis of variance (ANOVA) followed by Bonferroni's post hoc test to assess the differences between the treatment groups. Significant differences in comparison with those of the control group (vehicle-treated cells) are indicated by ^∗^*P* < 0.05; differences between LPS-treated and biphalin- or biphalin- and naloxone-treated cells are indicated by ^#^*P* < 0.05; differences between biphalin-treated and biphalin- and naloxone-treated cells are indicated by ^$^*P* < 0.05. (b) The presence of MOP in microglia was confirmed by double immunofluorescence. We found that MOP receptor (red) and OX-42 (a marker of microglial cells; green) were colocalized. The scale bar for all microphotographs is 25 *μ*m.

The level of MOP receptor was not modulated after LPS treatment compared to controls (Figure 8(a)). Biphalin pretreatment downregulated LPS-induced expression of MOP receptor protein (1.06 ± 0.05). Moreover, naloxone pretreatment changed the effect of biphalin (1.11 ± 0.12). No changes in the expression of MOP receptor were observed after treatment with compounds in vehicle-treated cells (Figure 8(a)).

Immunocytochemical localization of MOP receptors in primary microglial cells was confirmed by immunofluorescence (Figure 8(b)). By double immunofluorescence, we found that MOP receptor and OX-42 (a marker of microglial cells) were colocalized.

## 4. Discussion

Neuropathic pain is a vastly debilitating condition that adversely affects patients' quality of life. Our results show that biphalin reduced pain-related behavior in a preclinical model of neuropathy in rats, and these analgesic effects seem to be very important from a clinical point of view, especially because preclinical studies have revealed that biphalin has 1000-fold greater analgesic potency than morphine and induces less side effects [17, 18]. Moreover, our results provide strong evidence that biphalin, in contrast to morphine [32, 40, 41], is able to beneficially modulate crucial members of both microglial intracellular signaling pathways and proinflammatory factors, which are known to play a key role in neuropathic pain development.

Recently, it has been suggested that biphalin may be an alternative drug for use in cancer pain therapy due to its enhanced local analgesic activity and lower tolerance liability compared with morphine [13]; however, its beneficial properties in neuropathic pain have not been studied so far. Neuropathic pain is relatively less responsive to opioid therapy than other types of pain [1–5]. It has been suggested that the reduced effectiveness of opioids during neuropathy might be caused by faulty function of the spinal opioid system [4, 29, 37, 42, 43]. Lower morphine analgesia is also correlated with intense glutamatergic transmission [44–47] and increased levels of cholecystokinin [48], dynorphin [49], nociceptin [50], and melanocortin [51]. Recently, the changes in the function of nonneuronal cells, especially microglia, during neuropathic pain conditions were postulated to be the reason behind the disrupted actions of opioids [25, 27, 29, 30, 52]. It was shown that morphine signaling at microglial receptors might potentiate neuroinflammation [53, 54] and, moreover, that chronic morphine treatment leads to strong activation of glial cells and profound hyperalgesia and allodynia [6–9, 55]. It has also been shown that microglial cells possess MOP, KOP, and NOP, but not DOP, opioid receptors, which we confirmed at mRNA and protein levels using primary cell cultures [29, 30]. Our previous studies have shown that minocycline, an inhibitor of microglial activation [27, 30], strongly delayed the development of morphine tolerance [55, 56] and potentiated MOP, KOP, and NOP ligand analgesia [29, 30]. Several lines of evidence have revealed that morphine increases microglial reactivity [31, 57, 58] and promotes the proinflammatory phenotype of those cells [31, 32, 40]. Moreover, chronic morphine treatment leads to the development of hyperalgesia and allodynia [6–9].

In the present paper, we performed studies with biphalin, one of the most potent, peptide-based, opioid analgesics [59], which crosses the BBB to enter the CNS [60] and exerts less dependence and tolerance than morphine [17]. We are the first to reveal that biphalin reduced pain symptoms in CCI-exposed rats, and these analgesic effects in already ongoing pain processes seem to be very important from a clinical point of view. Moreover, we and others revealed that, during neuropathic pain, higher doses of morphine are required to obtain significant analgesic effects [1–5].

Several studies have revealed a beneficial role of biphalin on cell viability. In 2011, Yang et al. has shown that biphalin provides neuroprotection during stroke in an opioid receptor-dependent manner [61]. This dimeric opioid peptide also induced neuroprotective action on neurons in rat organotypic hippocampal cultures, which was abolished by naltrexone [62]. However, in our studies, biphalin did not evoke any significant changes in cell viability, both in vehicle- or LPS-treated microglial cells. Some authors have suggested that opioid-induced neuroprotection is mediated via DOP opioid receptors [63, 64]. Although those findings are in agreement with our data, we have previously shown that microglial cells do not possess DOP [29], and therefore, we suggest that biphalin mediates its effects by binding mainly to MOP receptors. Interestingly, it was shown that morphine causes significant decreases in microglial cell viability [41] at the same dose as biphalin used in our study. Moreover, in 2009, Horvath and DeLeo observed strong upregulation of Iba1 after morphine treatment (even in a nM doses), which was correlated with a promigratory phenotype of microglial cells. The effects of morphine on Iba1 protein level were mediated via MOP receptor [31]. Here, we have shown that biphalin did not affect Iba1 expression in vehicle-treated cells and, moreover, significantly diminished LPS-induced level of microglial activation marker in opioid-receptor dependent manner. Therefore, we suggest that biphalin actions are focused on the silencing of an excessive activation of microglia.

In the present study, we analyzed the effects of biphalin on crucial members of intracellular signaling pathways and on pro- and anti-inflammatory factors, which are known to play a role in neuropathic pain modulation. We indicated that biphalin significantly diminished LPS-induced NF-*κ*B activation in primary microglial cells by reducing phosphorylation of specific NF-*κ*B inhibitor, I*κ*B, in an opioid receptor-dependent manner. Moreover, this effect of biphalin was correlated with downregulation of proinflammatory factors, such as iNOS, IL-1*β*, IL-18, COX-2, and NLRP3. In the case of iNOS, IL-1*β*, and IL-18, the inhibitory action of biphalin was reversed by naloxone pretreatment; however, we observed a strong growing trend in COX-2 and NLRP3 expression after this antagonist blockade. As it was shown in many studies, NF-*κ*B is a crucial transcription factor, and its profound activation leads to neuropathic pain development and maintenance [25, 27, 65–68]. Our previously published results showed that inhibition of NF-*κ*B by its potent inhibitor, parthenolide, diminished symptoms of neuropathy, potentiated morphine analgesia, and diminished pronociceptive markers of microglial cell polarization (IL-1*β*, IL-18, and iNOS) [25, 37]. NF-*κ*B-dependent transcription of many proinflammatory factors, for example, NLRP3 inflammasomes [69, 70], whose function is crucial for the regulation of neuroinflammation mediated by microglia [71, 72], has also been documented. Subsequently, NLRP3 inflammasome is involved in IL-1*β* and IL-18 maturation [72]. Our results provide new evidence that biphalin more preferably affects microglia than morphine, which is known to enhance the release of IL-1*β*, TNF*α*, IL-6, and NO [32, 40] and activates NLRP3 inflammasome [70] in those cells.

In the present study, we also investigated the role of biphalin in modulating the STAT3 signaling pathway. Several lines of evidence have shown that STAT3 is an important participant in nociceptive transmission and microglial activation [25, 73–75]. Recent studies have examined that STAT3 is due to the polarization of the microglia/macrophages and may lead to the potentially neuroprotective “alternative activation” of those cells [25, 76–78]. However, this process depends on the type of activating factor, and it was already shown that LPS-stimulated microglial cells express high levels of STAT3 activation [75, 79]. Our study revealed that biphalin significantly diminishes the level of phosphorylated STAT3 and potentiates its inhibitor, SOCS3 protein, in LPS-stimulated cells. Moreover, biphalin diminishes upregulated levels of IL-6, IL-10, and TNF*α* but does not change the levels of IFN*γ* or TIMP-1. Przanowski and colleague showed that overexpression of active STAT3 protein in microglia leads to profound expression levels of IL-6, IL-10, and TNF*α* but not IFN*γ* [80]. Those data are in agreement with our present results, suggesting that IL-6, IL-10, and TNF*α* downregulation is mediated by biphalin-dependent STAT3 inhibition. The observed inhibitory effects of biphalin were not reversed by naloxone pretreatment, which suggests that this action is not mediated by opioid receptors. It is well documented that the analgesic efficacy of biphalin is in part due to its modest permeability of the blood-brain barrier [60, 81, 82]. In 1997, Romanowski et al. revealed that biphalin movement across the membrane is controlled by diffusion [82]. In 2015, Garbuz et al. demonstrated in a “tube test” assay (without any protein receptor presented) that biphalin has a strong antioxidant capability not related to opioid receptors [83]. Those reports confirmed that the biphalin actions we observed are not always related to opioid receptor signaling. Interestingly, the newest results obtained by Corder's group indicated that MOPs are expressed by nociceptors, but not microglia within the spinal cord [84]. Based on those results, our data explained well the strong antinociceptive effects of biphalin after intrathecal injection, since it is known that this opioid can act directly to the nociceptors and indirectly may modulate microglial-derived neuroinflammation in opioid receptor-independent manner.

Biphalin also differentially influences activation of the mitogen-activated protein kinase (MAPK), p38, and ERK1/2, other important participants in the intracellular signaling pathways that modulate the nociceptive response. The results obtained from animal studies revealed that p38 and ERK1/2 inhibition leads to a reduction of neuropathic pain symptoms and downregulation of pronociceptive factors [27, 37, 65, 85, 86]. In the present study, we showed that biphalin in opioid receptor manner downregulates p38 phosphorylation independent of those receptors inhibiting ERK1/2 activation. This pattern of the modulatory effects of biphalin suggests a cross-talk between p38-NF-*κ*B and ERK1/2-STAT3 in microglial-mediated neuroinflammation. In 2010, Wang and colleagues observed that tolerance to morphine-induced analgesia is regulated by activation of the microglial p38-NF-*κ*B and ERK-Stat1/3 cascade within astrocytes [87]. The results of our experiments suggest that the connection between these two pathways in microglia may also play an important role in neuroinflammation and pain progression, although this issue requires further study. Interestingly, others have discovered that, in contrast to biphalin, morphine significantly potentiates ERK1/2 [40], p38MAPK [41], NF-*κ*B, and I*κ*B [32] phosphorylation in microglia.

Several lines of evidence showed that microglial TLR, mainly subtype 4, plays a crucial role in the development of neuropathic pain [88–91]. Stimulation of TLR4 by invading pathogens or endogenous danger signals induces distinct patterns of gene expression, which leads to the microglia activation. Interestingly, it is suggested that opioids, for example, morphine, may bind to TLR4 and potentiate microglial proinflammatory phenotype [92]. However, this issue remains controversial and further investigations are needed to supplement the existing knowledge [33, 53, 93]. It is documented that endocysis and endosomal degradation of the LPS receptor complex is essential for signal termination [94, 95]. At first, we have shown that biphalin, in opioid-independent manner, restored TLR4-MyD88 pathway activation to control immunological response. Moreover, in 2016, Liang et al. demonstrated that morphine decreased TLR4 expression, and this receptor endocytosis was correlated with microglia activation [92]. Our data revealed that biphalin also controls LPS-induced MyD88-independent pathway, however, in opioid receptor-dependent manner. The data from TRIF knock-out mice demonstrated that TRIF deficiency diminished microglial cell activation and also limits the release of inflammatory cytokines following optic nerve injury [96], which correlated with our cell culture results showing that biphalin reduced TRIF expression to control level. Moreover, authors revealed [96] that NF-*κ*B activation in microglia is TRIF dependent, which is in agreement with our data, where biphalin modulates TRIF level, such as NF-*κ*B and p38MAPK. In 2009, Horvath and DeLeo showed that morphine promotes pronociceptive phenotype of microglial cells by MOP receptor [31]. Authors also observed increased expression of Iba1, which imparts a promigratory phenotype of microglia after morphine treatment in MOP receptor-dependent manner. Here, we observed that biphalin reduced expression of its classical opioid receptor, MOP, in activated microglia and also diminished Iba1 protein level in opioid receptor-dependent manner. Summing up, biphalin modulates TLR4-releated pathways and MOP receptor to restore the homeostasis of the cell and reduce microglia activation.

## 5. Conclusions

**Scheme 1:**
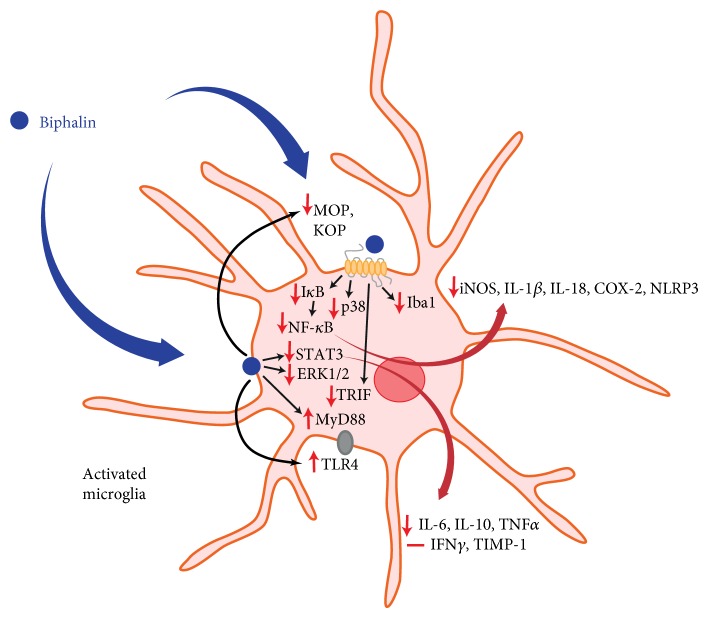
Hypothetical participation of biphalin in microglia-induced neuroinflammation. We suggest that the analgesic effects of biphalin during neuropathic pain are correlated with diminished microglia-induced neuroinflammation. Administration of biphalin reduces the activity of intracellular pathways in opioid receptor-dependent (NF-*κ*B, p38) and opioid receptor-independent (STAT3, ERK1/2) manners in primary cultures of microglia. Biphalin treatment reduces activation of the NF-*κ*B inhibitor, I*κ*B, and enhances the STAT3 inhibitor, SOCS3. In response to the modulation of intracellular pathways by biphalin, the production of pronociceptive factors is diminished (iNOS, IL-1*β*, IL-18, COX-2, NLPR3, IL-6, and TNF*α*). Biphalin diminishes expression of microglial marker of cell activation (Iba1). Biphalin also modulates TLR4-related pathways, as well attenuates MOP receptor level to restore the homeostasis of the cell and reduce microglia activation. Our present data, as well as earlier reports [13, 17, 18], strongly suggest that biphalin can be considered as a promising therapeutic agent for the treatment of pain and other CNS pathologies correlated with neuroinflammation. Abbreviations: MOP, mu opioid receptor; DOP, delta opioid receptor; KOP, kappa opioid receptor; ERK1/2, extracellular signal-regulated kinase 1/2; STAT3, signal transducers and activators of transcription 3; SOCS3, suppressor of cytokine signaling 3; NF-*κ*B, nuclear factor-*κ*B; I*κ*B, inhibitor of nuclear factor-*κ*B; iNOS, inducible nitric oxide synthase; IL, interleukin; NLRP3, nucleotide-binding domain- (NOD-) like receptor protein 3; COX-2, cyclooxygenase 2; TNF*α*, tumor necrosis factor *α*; IFN*γ*, interferon *γ*; Iba1, ionized calcium-binding adaptor molecule 1; TLR4, Toll-like receptor 4; MyD88, myeloid differentiation primary response gene 88; and TRIF, TIR-domain-containing adapter-inducing interferon-*β*.

Dysregulation of microglial cells may result in pathological conditions of the CNS, such as neuropathic pain, and may diminish the analgesic action of opioids. We revealed that biphalin exerts beneficial effects during ongoing neuropathic pain. The results of the present study also revealed that the analgesic effects of biphalin not only are correlated with activation of the neuronal pool of opioid receptors but may also be correlated with diminished microglial-induced neuroinflammation (Scheme 1). In the light of the newest results concerning the lack of MOP receptors on microglia within the spinal cord [84], our studies provide an evidence that analgesic effects of biphalin might also be mediated in opioid receptor-independent manner. Our present data, as well as earlier reports, strongly suggest that biphalin can be considered as a promising therapeutic agent for the treatment of pain and other CNS pathologies correlated with neuroinflammation. We also provide a strong evidence that profound biphalin-induced analgesia, contrary to morphine [31, 32, 40, 41, 92], is correlated with diminished microglia-induced inflammation.

## Abbreviations

BIPH: Biphalin
CCI: Chronic constriction injury
CNS: Central nervous system
COX-2: Cyclooxygenase 2
DOP: Delta opioid receptor
ERK1/2: Extracellular signal-regulated kinase 1/2
Iba1: Ionized calcium-binding adaptor molecule 1
IFN*γ*: Interferon *γ*
IL: Interleukin
iNOS: Inducible nitric oxide synthase
I*κ*B: Inhibitor of nuclear factor-*κ*B
KOP: Kappa opioid receptor
LPS: Lipopolysaccharide
MOP: Mu opioid receptor
MyD88: Myeloid differentiation primary response gene 88
NF-*κ*B: Nuclear factor-*κ*B
NLRP3: Nucleotide-binding domain- (NOD-) like receptor protein 3
NLX: Naloxone
SOCS3: Suppressor of cytokine signaling 3
STAT3: Signal transducers and activators of transcription 3
TLR4: Toll-like receptor 4
TNF*α:*: Tumor necrosis factor *α*
TRIF: TIR-domain-containing adapter-inducing interferon-*β*.

## References

[1] Arnér S., Meyerson B. (1988). Lack of analgesic effect of opioids on neuropathic and idiopathic forms of pain. *Pain*.

[2] Eisenberg E., McNicol E. D., Carr D. B. (2005). Efficacy and safety of opioid agonists in the treatment of neuropathic pain of nonmalignant origin: systematic review and meta-analysis of randomized controlled trials. *Journal of the American Medical Association*.

[3] Hirsch S. J., Dickenson A. H. (2014). Morphine sensitivity of spinal neurons in the chronic constriction injury neuropathic rat pain model. *Neuroscience Letters*.

[4] Obara I., Parkitna J., Korostynski M. (2009). Local peripheral opioid effects and expression of opioid genes in the spinal cord and dorsal root ganglia in neuropathic and inflammatory pain. *Pain*.

[5] Przewlocki R., Przewlocka B. (2005). Opioids in neuropathic pain. *Current Pharmaceutical Design*.

[6] Beitner-Johnson D., Guitart X., Nestler E. J. (1993). Glial fibrillary acidic protein and the mesolimbic dopamine system: regulation by chronic morphine and Lewis-Fischer strain differences in the rat ventral tegmental area. *Journal of Neurochemistry*.

[7] Ferrini F., Trang T., Mattioli T.-A. M. (2013). Morphine hyperalgesia gated through microglia-mediated disruption of neuronal Cl^−^ homeostasis. *Nature Neuroscience*.

[8] Loram L. C., Grace P. M., Strand K. A. (2012). Prior exposure to repeated morphine potentiates mechanical allodynia induced by peripheral inflammation and neuropathy. *Brain, Behavior, and Immunity*.

[9] Tumati S., Largent-Milnes T., Keresztes A. (2012). Repeated morphine treatment-mediated hyperalgesia, allodynia and spinal glial activation are blocked by co-administration of a selective cannabinoid receptor type-2 agonist. *Journal of Neuroimmunology*.

[10] Lipkowski A., Carr D., Silbert B., Cepeda M., Osgood P., Szyfelbein S. (1994). Non-deterministic individual responses to receptor-selective opioid agonists. *Polish Journal of Pharmacology*.

[11] Lipkowski A., Konecka A. M., Sroczynska I., Przewlocki R., Stala L., Tam S. W. (1987). Bivalent opioid peptide analogues with reduced distances between pharmacophores. *Life Sciences*.

[12] Slaninova J., Appleyard S. M., Misicka A. (1998). [125I-Tyr1]biphalin binding to opioid receptors of rat brain and NG108-15 cell membranes. *Life Sciences*.

[13] Lesniak A., Bochynska-Czyz M., Sacharczuk M. (2016). Biphalin preferentially recruits peripheral opioid receptors to facilitate analgesia in a mouse model of cancer pain—a comparison with morphine. *European Journal of Pharmaceutical Sciences*.

[14] Sobczak M., Pilarczyk A., Jonakowski M. (2014). Anti-inflammatory and antinociceptive action of the dimeric enkephalin peptide biphalin in the mouse model of colitis: new potential treatment of abdominal pain associated with inflammatory bowel diseases. *Peptides*.

[15] Huber J. D., Campos C. R., Egleton R. D. (2003). Conjugation of low molecular weight poly(ethylene glycol) to biphalin enhances antinociceptive profile. *Journal of Pharmaceutical Sciences*.

[16] Silbert B. S., Lipkowski A. W., Cepeda M. S., Szyfelbein S. K., Osgood P. F., Carr D. B. (1991). Analgesic activity of a novel bivalent opioid peptide compared to morphine via different routes of administration. *Agents and Actions*.

[17] Yamazaki M., Suzuki T., Narita M., Lipkowski A. W. (2001). The opioid peptide analogue biphalin induces less physical dependence than morphine. *Life Sciences*.

[18] Kosson D., Klinowiecka A., Kosson P. (2008). Intrathecal antinociceptive interaction between the NMDA antagonist ketamine and the opioids, morphine and biphalin. *European Journal of Pain*.

[19] Boddeke E. (2001). Involvement of chemokines in pain. *European Journal of Pharmacology*.

[20] Johnston I., Milligan E., Wieseler-Frank J. (2004). A role for proinflammatory cytokines and fractalkine in analgesia, tolerance, and subsequent pain facilitation induced by chronic intrathecal morphine. *The Journal of Neuroscience*.

[21] Malek N., Popiolek-Barczyk K., Mika J., Przewlocka B., Starowicz K. (2015). Anandamide, acting via *CB2* receptors, alleviates LPS-induced neuroinflammation in rat primary microglial cultures. *Neural Plasticity*.

[22] Mika J., Zychowska M., Popiolek-Barczyk K., Rojewska E., Przewlocka B. (2013). Importance of glial activation in neuropathic pain. *European Journal of Pharmacology*.

[23] Oprée A., Kress M. (2000). Involvement of the proinflammatory cytokines tumor necrosis factor-alpha, IL-1 beta, and IL-6 but not IL-8 in the development of heat hyperalgesia: effects on heat-evoked calcitonin gene-related peptide release from rat skin. *The Journal of Neuroscience*.

[24] Pilat D., Rojewska E., Jurga A. M. (2015). IL-1 receptor antagonist improves morphine and buprenorphine efficacy in a rat neuropathic pain model. *European Journal of Pharmacology*.

[25] Popiolek-Barczyk K., Kolosowska N., Piotrowska A. (2015). Parthenolide relieves pain and promotes M2 microglia/macrophage polarization in rat model of neuropathy. *Neural Plasticity*.

[26] Slusarczyk J., Trojan E., Glombik K. (2015). Prenatal stress is a vulnerability factor for altered morphology and biological activity of microglia cells. *Frontiers in Cellular Neuroscience*.

[27] Rojewska E., Popiolek-Barczyk K., Jurga A. M., Makuch W., Przewlocka B., Mika J. (2014). Involvement of pro- and antinociceptive factors in minocycline analgesia in rat neuropathic pain model. *Journal of Neuroimmunology*.

[28] DeLeo J., Yezierski R. (2001). The role of neuroinflammation and neuroimmune activation in persistent pain. *Pain*.

[29] Mika J., Popiolek-Barczyk K., Rojewska E., Makuch W., Starowicz K., Przewlocka B. (2014). Delta-opioid receptor analgesia is independent of microglial activation in a rat model of neuropathic pain. *PloS One*.

[30] Popiolek-Barczyk K., Rojewska E., Jurga A. (2014). Minocycline enhances the effectiveness of nociceptin/orphanin FQ during neuropathic pain. *BioMed Research International*.

[31] Horvath R. J., DeLeo J. A. (2009). Morphine enhances microglial migration through modulation of P2X4 receptor signaling. *The Journal of Neuroscience*.

[32] Gessi S., Borea P. A., Bencivenni S., Fazzi D., Varani K., Merighi S. (2016). The activation of μ-opioid receptor potentiates LPS-induced NF-kB promoting an inflammatory phenotype in microglia. *FEBS Letters*.

[33] Wang X., Loram L. C., Ramos K. (2012). Morphine activates neuroinflammation in a manner parallel to endotoxin. *Proceedings of the National Academy of Sciences*.

[34] Zimmermann M. (1983). Ethical guidelines for investigations of experimental pain in conscious animals. *Pain*.

[35] Yaksh T. L., Rudy T. A. (1976). Chronic catheterization of the spinal subarachnoid space. *Physiology & Behavior*.

[36] Bennett G. J., Xie Y. K. (1988). A peripheral mononeuropathy in rat that produces disorders of pain sensation like those seen in man. *Pain*.

[37] Popiolek-Barczyk K., Makuch W., Rojewska E., Pilat D., Mika J. (2014). Inhibition of intracellular signaling pathways NF-κB and MEK1/2 attenuates neuropathic pain development and enhances morphine analgesia. *Pharmacological Reports*.

[38] Campbell J., Raja S., Meyer R., Mackinnon S. (1988). Myelinated afferents signal the hyperalgesia associated with nerve injury. *Pain*.

[39] Zawadzka M., Kaminska B. (2005). A novel mechanism of FK506-mediated neuroprotection: downregulation of cytokine expression in glial cells. *Glia*.

[40] Merighi S., Gessi S., Varani K., Fazzi D., Stefanelli A., Borea P. A. (2013). Morphine mediates a proinflammatory phenotype via m-opioid receptor-PKCeps-Akt-ERK1/2 signaling pathway in activated microglial cells. *Biochemical Pharmacology*.

[41] Xie N., Li H., Wei D. (2010). Glycogen synthase kinase-3 and p38 MAPK are required for opioid-induced microglia apoptosis. *Neuropharmacology*.

[42] Bian D., Nichols M. L., Ossipov M. H., Lai J., Porreca F. (1995). Characterization of the antiallodynic efficacy of morphine in a model of neuropathic pain in rats. *Neuroreport*.

[43] Ossipov M. H., Lopez Y., Nichols M. L., Bian D., Porreca F. (1995). The loss of antinociceptive efficacy of spinal morphine in rats with nerve ligation injury is prevented by reducing spinal afferent drive. *Neuroscience Letters*.

[44] Bal-Price A., Brown G. C. (2001). Inflammatory neurodegeneration mediated by nitric oxide from activated glia-inhibiting neuronal respiration, causing glutamate release and excitotoxicity. *The Journal of Neuroscience*.

[45] Osikowicz M., Mika J., Makuch W., Przewlocka B. (2008). Glutamate receptor ligands attenuate allodynia and hyperalgesia and potentiate morphine effects in a mouse model of neuropathic pain. *Pain*.

[46] Osikowicz M., Skup M., Mika J., Makuch W., Czarkowska-Bauch J., Przewlocka B. (2009). Glial inhibitors influence the mRNA and protein levels of mGlu2/3, 5 and 7 receptors and potentiate the analgesic effects of their ligands in a mouse model of neuropathic pain. *Pain*.

[47] Popik P., Kozela E., Pilc A. (2000). Selective agonist of group II glutamate metabotropic receptors, LY354740, inhibits tolerance to analgesic effects of morphine in mice. *British Journal of Pharmacology*.

[48] Stanfa L., Dickenson A., Xu X., Wiesenfeld-Hallin Z. (1994). Cholecystokinin and morphine analgesia: variations on a theme. *Trends in Pharmacological Sciences*.

[49] Obara I., Mika J., Schafer M., Przewlocka B. (2003). Antagonists of the kappa-opioid receptor enhance allodynia in rats and mice after sciatic nerve ligation. *British Journal of Pharmacology*.

[50] Mika J., Schafer M. K., Obara I., Weihe E., Przewlocka B. (2004). Morphine and endomorphin-1 differently influence pronociceptin/orphanin FQ system in neuropathic rats. *Pharmacology, Biochemistry, and Behavior*.

[51] Starowicz K., Obara I., Przewlocki R., Przewlocka B. (2005). Inhibition of morphine tolerance by spinal melanocortin receptor blockade. *Pain*.

[52] Watkins L. R., Hutchinson M. R., Johnston I. N., Maier S. F. (2005). Glia: novel counter-regulators of opioid analgesia. *Trends in Neurosciences*.

[53] Hutchinson M. R., Zhang Y., Shridhar M. (2010). Evidence that opioids may have toll-like receptor 4 and MD-2 effects. *Brain, Behavior, and Immunity*.

[54] Lewis S. S., Hutchinson M. R., Rezvani N. (2010). Evidence that intrathecal morphine-3-glucuronide may cause pain enhancement via toll-like receptor 4/MD-2 and interleukin-1β. *Neuroscience*.

[55] Mika J., Wawrzczak-Bargiela A., Osikowicz M., Makuch W., Przewlocka B. (2009). Attenuation of morphine tolerance by minocycline and pentoxifylline in naive and neuropathic mice. *Brain, Behavior, and Immunity*.

[56] Mika J., Osikowicz M., Makuch W., Przewlocka B. (2007). Minocycline and pentoxifylline attenuate allodynia and hyperalgesia and potentiate the effects of morphine in rat and mouse models of neuropathic pain. *European Journal of Pharmacology*.

[57] Raghavendra V., Rutkowski M. D., DeLeo J. A. (2002). The role of spinal neuroimmune activation in morphine tolerance/hyperalgesia in neuropathic and sham-operated rats. *The Journal of Neuroscience*.

[58] Tawfik V. L., LaCroix-Fralish M. L., Nutile-McMenemy N., DeLeo J. A. (2005). Transcriptional and translational regulation of glial activation by morphine in a rodent model of neuropathic pain. *The Journal of Pharmacology and Experimental Therapeutics*.

[59] Feliciani F., Pinnen F., Stefanucci A. (2013). Structure-activity relationships of biphalin analogs and their biological evaluation on opioid receptors. *Mini Reviews in Medicinal Chemistry*.

[60] Horan P. J., Mattia A., Bilsky E. J. (1993). Antinociceptive profile of biphalin, a dimeric enkephalin analog. *The Journal of Pharmacology and Experimental Therapeutics*.

[61] Yang L., Shah K., Wang H., Karamyan V. T., Abbruscato T. J. (2011). Characterization of neuroprotective effects of biphalin, an opioid receptor agonist, in a model of focal brain ischemia. *The Journal of Pharmacology and Experimental Therapeutics*.

[62] Kawalec M., Kowalczyk J. E., Beresewicz M., Lipkowski A. W., Zablocka B. (2011). Neuroprotective potential of biphalin, multireceptor opioid peptide, against excitotoxic injury in hippocampal organotypic culture. *Neurochemical Research*.

[63] Ma M. C., Qian H., Ghassemi F., Zhao P., Xia Y. (2005). Oxygen-sensitive delta-opioid receptor-regulated survival and death signals: novel insights into neuronal preconditioning and protection. *The Journal of Biological Chemistry*.

[64] Zhang J., Haddad G. G., Xia Y. (2000). Delta-, but not mu- and kappa-, opioid receptor activation protects neocortical neurons from glutamate-induced excitotoxic injury. *Brain Research*.

[65] Rojewska E., Popiolek-Barczyk K., Kolosowska N. (2015). PD98059 influences immune factors and enhances opioid analgesia in model of neuropathy. *PloS One*.

[66] Miyoshi K., Obata K., Kondo T., Okamura H., Noguchi K. (2008). Interleukin-18-mediated microglia/astrocyte interaction in the spinal cord enhances neuropathic pain processing after nerve injury. *The Journal of Neuroscience*.

[67] Meunier A., Latrémolière A., Dominguez E., Ther M. (2007). Lentiviral-mediated targeted NF- κ B blockade in dorsal spinal cord glia attenuates sciatic nerve injury—induced neuropathic pain in the rat.

[68] Ma W., Bisby M. (1998). Increased activation of nuclear factor kappa B in rat lumbar dorsal root ganglion neurons following partial sciatic nerve injuries. *Brain Research*.

[69] Lamkanfi M., Dixit V. M. (2009). The inflammasomes. *PLoS Pathogens*.

[70] Cai Y., Kong H., Pan Y.-B. (2016). Procyanidins alleviates morphine tolerance by inhibiting activation of NLRP3 inflammasome in microglia. *Journal of Neuroinflammation*.

[71] Gustin A., Kirchmeyer M., Koncina E. (2015). NLRP3 inflammasome is expressed and functional in mouse brain microglia but not in astrocytes. *PloS One*.

[72] Hanamsagar R., Torres V., Kielian T. (2011). Inflammasome activation and IL-1b/IL-18 processing are influenced by distinct pathways in microglia. *Journal of Neurochemistry*.

[73] Dominguez E., Rivat C., Pommier B., Mauborgne A., Pohl M. (2008). JAK/STAT3 pathway is activated in spinal cord microglia after peripheral nerve injury and contributes to neuropathic pain development in rat. *Journal of Neurochemistry*.

[74] Dominguez E., Mauborgne A., Mallet J., Desclaux M., Pohl M. (2010). SOCS3-mediated blockade of JAK/STAT3 signaling pathway reveals its major contribution to spinal cord neuroinflammation and mechanical allodynia after peripheral nerve injury. *The Journal of Neuroscience*.

[75] Piotrowska A., Kwiatkowski K., Rojewska E., Makuch W., Mika J. (2016). Maraviroc reduces neuropathic pain through polarization of microglia and astroglia—evidence from in vivo and in vitro studies. *Neuropharmacology*.

[76] Koscsó B., Csóka B., Kókai E. (2013). Adenosine augments IL-10-induced STAT3 signaling in M2c macrophages. *Journal of Leukocyte Biology*.

[77] Cianciulli A., Dragone T., Calvello R. (2015). IL-10 plays a pivotal role in anti-inflammatory effects of resveratrol in activated microglia cells. *International Immunopharmacology*.

[78] Popiolek-Barczyk K., Mika J. (2016). Targeting the microglial signaling pathways: new insights in the modulation of neuropathic pain. *Current Medicinal Chemistry*.

[79] Tam W. Y., Ma C. H. E. (2014). Bipolar/rod-shaped microglia are proliferating microglia with distinct M1/M2 phenotypes. *Scientific Reports*.

[80] Przanowski P., Dabrowski M., Ellert-Miklaszewska A. (2014). The signal transducers Stat1 and Stat3 and their novel target Jmjd3 drive the expression of inflammatory genes in microglia. *Journal of Molecular Medicine*.

[81] Abbruscato T. J., Williams S. A., Misicka A., Lipkowski A. W., Hruby V. J., Davis T. P. (1996). Blood-to-central nervous system entry and stability of biphalin, a unique double-enkephalin analog, and its halogenated derivatives. *The Journal of Pharmacology and Experimental Therapeutics*.

[82] Romanowski M., Zhu X., Ramaswami V. (1997). Interaction of a highly potent dimeric enkephalin analog, biphalin, with model membranes. *Biochimica et Biophysica Acta*.

[83] Garbuz O., Gulea A., Dyniewicz J., Zablocka B., Lipkowski A. W. (2015). The non-opioid receptor, antioxidant properties of morphine and the opioid peptide analog biphalin. *Peptides*.

[84] Corder G., Tawfik V., Wang D. (2017). Loss of μ opioid receptor signaling in nociceptors, but not microglia, abrogates morphine tolerance without disrupting analgesia. *Nature Medicine*.

[85] Tsuda M., Mizokoshi A., Shigemoto-Mogami Y., Koizumi S., Inoue K. (2004). Activation of p38 mitogen-activated protein kinase in spinal hyperactive microglia contributes to pain hypersensitivity following peripheral nerve injury. *Glia*.

[86] Jin S.-X., Zhuang Z.-Y., Woolf C. J., Ji R.-R. (2003). P38 mitogen-activated protein kinase is activated after a spinal nerve ligation in spinal cord microglia and dorsal root ganglion neurons and contributes to the generation of neuropathic pain. *The Journal of Neuroscience*.

[87] Wang Z., Ma W., Chabot J. G., Quirion R. (2010). Calcitonin gene-related peptide as a regulator of neuronal CaMKII-CREB, microglial p38-NFκB and astroglial ERK-Stat1/3 cascades mediating the development of tolerance to morphine-induced analgesia. *Pain*.

[88] Lehnardt S., Massillon L., Follett P. (2003). Activation of innate immunity in the CNS triggers neurodegeneration through a Toll-like receptor 4-dependent pathway. *Proceedings of the National Academy of Sciences of the United States of America*.

[89] Jurga A. M., Rojewska E., Piotrowska A. (2016). Blockade of toll-like receptors (TLR2, TLR4) attenuates pain and potentiates buprenorphine analgesia in a rat neuropathic pain model. *Neural Plasticity*.

[90] Tanga F. Y., Nutile-McMenemy N., DeLeo J. A. (2005). The CNS role of Toll-like receptor 4 in innate neuroimmunity and painful neuropathy. *Proceedings of the National Academy of Sciences of the United States of America*.

[91] Kim D., Kim M. A., Cho I. H. (2007). A critical role of toll-like receptor 2 in nerve injury-induced spinal cord glial cell activation and pain hypersensitivity. *The Journal of Biological Chemistry*.

[92] Liang Y., Chu H., Jiang Y., Yuan L. (2016). Morphine enhances IL-1 β release through toll-like receptor 4-mediated endocytic pathway in microglia. *Purinergic Signal*.

[93] Grace P. M., Maier S. F., Watkins L. R. (2015). Opioid-induced central immune signaling: implications for opioid analgesia. *Headache*.

[94] Latz E., Visintin A., Lien E. (2002). Lipopolysaccharide rapidly traffics to and from the Golgi apparatus with the toll-like receptor 4-MD-2-CD14 of signal transduction. *The Journal of Biological Chemistry*.

[95] Thieblemont N., Wright S. D. (1999). Transport of bacterial lipopolysaccharide to the Golgi apparatus. *The Journal of Experimental Medicine*.

[96] Lin S., Liang Y., Zhang J. (2012). Microglial TIR-domain-containing adapter- inducing interferon- b ( TRIF ) deficiency promotes retinal ganglion cell survival and axon regeneration via nuclear factor- B. *Journal of Neuroinflammation*.

